# Assessing a Multilayered Hydrophilic–Electrocatalytic Forward Osmosis Membrane for Ammonia Electro-Oxidation

**DOI:** 10.3390/membranes15020037

**Published:** 2025-01-22

**Authors:** Perla Cruz-Tato, Laura I. Penabad, César Lasalde, Alondra S. Rodríguez-Rolón, Eduardo Nicolau

**Affiliations:** 1 Department of Chemistry, University of Puerto Rico—Río Piedras Campus, 17 University Ave. 1701, San Juan, PR 00925, USA; perla.cruz@upr.edu (P.C.-T.); alondra.rodriguez4@upr.edu (A.S.R.-R.); 2 Molecular Sciences Research Center, 1390 Ponce De Leon Ave., Suite 2, San Juan, PR 00931, USA; 3 Department of Chemistry, University of Michigan, 930 N University Ave., Ann Arbor, MI 48109, USA; penabadp@umich.edu; 4 Department of Applied Physics and Materials Science, California Institute of Technology, 1200 E California Blvd., Pasadena, CA 91125, USA; clasalde@caltech.edu

**Keywords:** carbon black, forward osmosis, electrocatalytic membrane, hydrophilic, ammonia electro-oxidation

## Abstract

Over the years, the ammonia concentration in water streams and the environment is increasing at an alarming rate. Many membrane-based processes have been studied to alleviate this concern via adsorption and filtration. On the other hand, ammonia electro-oxidation is an approach of particular interest owing to its energetic and environmental benefits. Thus, a plausible alternative to combine these two paths is by using an electroconductive membrane (ECM) to complete the ammonia oxidation reaction (AOR). This combination of processes has been studied very limitedly, and it can be an area for development. Herein, we developed a multilayered membrane with hydrophilic and electrocatalytic properties capable of completing the AOR. The porosity of carbon black (CB) particles was embedded in the polymeric support (CBES) and the active side was composed of a triple layer consisting of polyamide/CB/Pt nanoparticles (PA:CB:Pt). The CBES increased the membrane porosity, changed the pores morphology, and enhanced water permeability and electroconductivity. The deposition of each layer was monitored and corroborated physically, chemically, and electrochemically. The final membrane CBES:PA:VXC:Pt reached higher water flux than its PSF counterpart (3.9 ± 0.3 LMH), had a hydrophilic surface (water contact angle: 19.8 ± 0.4°), and achieved the AOR at −0.3 V vs. Ag/AgCl. Our results suggest that ECMs with conductive material in both membrane layers enhanced their electrical properties. Moreover, this study is proof-of-concept that the AOR can be succeeded by a polymeric FO-ECMs.

## 1. Introduction

The production of ammonia has increased remarkably in recent years due to its diverse uses, e.g., fertilizer, antimicrobial agents, as a precursor for nitrogenous compounds, among others. This leads to a large fraction being released into the environment, including domestic sewage discharges. Therefore, ammonia removal from water/wastewater has gained significant attention, and several technologies/methods have been investigated [1,2,3,4]. Among these, membrane-based technologies have been explored, however, the main focus has been the membrane’s adsorptive capacity [5], size-exclusion [6] or chemical-exclusion [7] properties, and gas extraction ability [8].

Ammonia oxidation reaction (AOR) is one prominent electrochemical reaction with great potential to remove ammonia from contaminated water but also to contribute to environmental applications (e.g., ammonia fuel cells). Yet the sluggish reaction kinetics require the use of electrocatalysts, and platinum is considered one of the most active [9,10,11]. Most researchers have been centering their efforts on developing cost-effective and selective catalysts [4]. One approach that can be implemented is the use of electroconductive membranes (ECMs) as the catalyst to conduct the AOR. ECMs are a class of membranes where an electroactive additive is incorporated into at least one of the membrane’s layers without affecting water permeability. There has been plenty of evolution in this research area [12], where ECMs are capable to prevent and mitigate the adhesion of foulants via physical [13] (i.e., electrostatic repulsions) and chemical [14] (i.e., degradation reactions) processes. However, the AOR using ECMs for filtration applications has not been explored, thus, it is still an area for development.

ECMs have proven to be a successful approach where the membrane combines its selective separation potential with an electrochemical process. Amongst the ECMs, different types of technologies have been investigated, including ultrafiltration [15], nanofiltration [16], and reverse osmosis [17]. Less attention has been paid to osmotically-driven ECMs owing to the differences in water permeability rates. Nonetheless, we focused on the forward osmosis (FO) process because their separation efficiency is comparable to that of the pressure-driven processes, while the energy input for the driving force (i.e., osmotic potential gradient) is substantially reduced.

Typically, FO membranes are composed of two layers: a porous support and an active layer. Studies have shown that modifications to any of these layers can modify the membrane properties. An ideal FO-ECM to conduct the AOR should exhibit a hydrophilic surface to attract the ammonia molecules while allowing the water molecules to permeate through the pores and an ammonia-selective catalytic surface. It is known that hydrophilic surfaces can create a hydration layer, some reported examples are the use of amine-based polymers [18,19] and zwitterions [20]. Currently, for the electroconductive character, refined and highly graphitized carbonaceous species like carbon nanotubes and graphene oxides have been studied [21]. In contrast, carbon black (CB) particles are less refined conductive porous particles widely studied as electrocatalyst substrates [22,23] but are rarely used in the membrane research area. CBs are composed of sp^2^ carbons with defect sites (sp^3^ carbons) covalently bonded to hydrophilic functional groups, such as epoxy, –COOH, and –OH [24]. Some membranes containing CBs have emphasized their capacity to enhance the conductivity [25] and good chemical adhesion with polymeric materials [26,27]. Also, CBs have been used in polymer electrolyte membrane fuel cells (PEMFCs) to stabilize and minimize the electrocatalyst agglomeration [28]. Similarly, these CB particles could enhance the mechanical and electrochemical properties of a water filtration membrane.

As a feasible alternative, we developed a multilayered membrane exhibiting hydrophilic and electrocatalytic properties as a potential FO-ECM for the AOR. The rationale for designing this complex layered membrane was to integrate distinct properties while allowing the materials to operate synergistically. To do so, we designed the following composite membrane: (L1) polymeric support with CBs particles, (L2) polyamide (PA), a dense hydrophilic polymer, (L3) Vulcan XC-72 (VXC), a type of CBs with high surface area and electroconductivity, and (L4) platinum nanoparticles as electrocatalyst for the AOR. We monitored the morphological changes by all the layer deposition (L1–L4) using SEM, the electrochemical properties were validated using CV and EIS, and the wettability of the membrane’s active layer was studied with contact angle measurements. The FO performance was examined after the deposition of each layer. Lastly, the AOR was assessed by CV and chronoamperometry. To the best of our knowledge, this is the first time this combination of materials has been explored for a FO-ECM. This work represents the successful development of a FO-ECM tailored for AOR. It provides valuable insights into the effect of integrating materials with different properties in both layers of the membrane (i.e., support and active) to reach a dual-functional FO membrane. More importantly, this study opens new possibilities for ECM and highlights their potential for ammonia removal from water and wastewater.

## 2. Materials and Methods

### 2.1. Materials

Polysulfone (PSF, average Mn ~22,000), N-methyl-2-pyrrolidone (NMP, 99%), m-phenylenediamine (MPD, 99%), trimesoyl chloride (TMC, 98%), hexane (anhydrous 95%), sodium carbonate (Na_2_CO_3_, ACS reagent 99%), sodium dodecylbenzenesulfonate (SDBS, technical grade), poly(vinyl alcohol) (PVA 150 K), glutaraldehyde (GA, 50 wt. % in H_2_O), hydrochloric acid (HCl, ACS reagent 37%), potassium tetrachloroplatinate (K_2_PtCl_4_, 99.99%), sulfuric acid (H_2_SO_4_, ACS reagent 95.0–98.0%), sodium chloride (NaCl, ACS reagent 99.0%), potassium hydroxide (KOH, ACS reagent, ≥85%, pellets), potassium chloride (KCl, ACS reagent 99%), potassium ferrocyanide trihydrate (K_4_[Fe(CN)_6_], ACS reagent, 99%), and potassium ferricyanide (K_3_[Fe(CN)_6_], ACS reagent, 99%) were all purchased from Sigma-Aldrich (Burlington, MA, USA). Carbon black: Vulcan XC-72 (VXC), Black Pearl 280 (BP2), and Black Pearl 430 (BP4) were obtained from Cabot Corp (Boston, MA, USA). Polyester mesh (105 micron—52% open area) was purchased from Elko Filtering Co (Fort Lauderdale, FL, USA). All chemicals and solvents were used as received without further purification. Nanopure water (18.2 MΩ·cm^2^) was used at all times.

### 2.2. Methods

#### 2.2.1. Multilayered Membrane Preparation (See Scheme 1)

##### Polymeric Support (Layer 1)

The polymeric supports were prepared via the non-solvent induced phase separation (NIPS) process. Briefly, two solutions: (a) 12% *w*/*v* of polysulfone (PSF) and (b) 12% PSF and 5% *w*/*v* black pearls 480 (BP4) were dissolved in N-methyl-2-pyrrolidone (NMP) overnight. These solutions were casted over a polyester mesh adjusting the thickness to 150 µm. Then, the respective films were immersed in a nanopure water (npw) precipitation bath and allowed at least 10 min to complete the precipitation process. The two supports: (a) PSF and (b) PSF-BP4, carbon black embedded support (CBES) were stored in npw.

##### Polyamide—Hydrophilic Layer (Layer 2)

Once the supports were fabricated, a 2% *w*/*v* m-phenylenediamine (MPD) aqueous solution was poured onto the membrane’s surface for 2 min and the excess was removed using an air knife. Next, a 0.1% *w*/*v* trimesoyl chloride (TMC) solution in hexane was poured onto the membrane’s surface for 1 min and the excess was removed by using an air knife. Finally, the membranes were soaked in a 0.2% *w*/*v* sodium carbonate solution for 5 min, rinsed, and stored in npw.

##### Carbon Black Vulcan—Conductive Layer (Layer 3)

An aqueous solution of 1% Vulcan (VXC) and 10% sodium dodecylbenzenesulfonate (SDBS) surfactant was prepared and sonicated until complete dissolution. Thereafter, this solution was spray coated over the X:PA membranes (where X = PSF or CBES) at a concentration of 0.05 mL/cm^2^, maintaining a 3 inch distance between the nozzle and the membrane. The coating was left to dry at room temperature. An aqueous solution of 1% poly(vinyl alcohol) (PVA) was prepared, where the PVA was dissolved by stirring in npw at 100 °C. Then, a thin layer of the PVA solution was air-sprayed over the VXC-SDBS coating. After drying at room temperature, the membranes were rinsed with abundant npw to remove the SDBS. Lastly, to complete the crosslinking reaction, the membranes were immersed in a 1% glutaraldehyde/1% HCl solution at 75 °C for 1 h. Once the reaction was completed, the modified membranes (X:PA:VXC) were rinsed with abundant npw and stored in water until used.

##### Platinum Nanoparticles Electrodeposition (Layer 4)

Once the CB conductive layer was deposited, platinum nanoparticles were electrodeposited using a Biologic SP-240 Potentiostat from Biologic USA (Knoxville, TN, USA) and a custom-made 3-electrode cell [29]. The membrane was used as the working electrode on a fluorine-doped tin oxide (FTO) and carbon tape contact, the reference electrode was Ag/AgCl (0.197V vs. NHE), and the counter electrode was a platinum wire. Cyclic voltammetry (CV) was used for the electrodeposition with a potential window from −0.6 V to 1.4 V vs. Ag/AgCl in 5 mM K_2_PtCl_4_ in 0.5 M H_2_SO_4_ at a scan rate of 50 mV/s for 50 cycles. Once the reaction was completed, the membrane was rinsed thoroughly with npw.

**Scheme 1:**
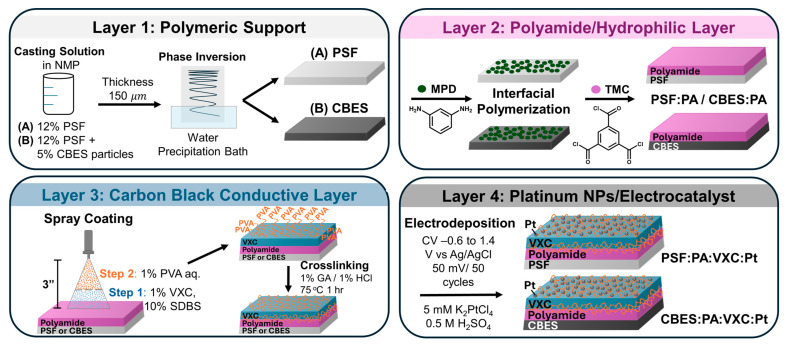
Representation of the membrane assembling steps. L1: the support was manufactured via phase inversion with two different casting solutions, L2: interfacial polymerization of polyamide film, L3: spray coating of VXC, and PVA followed by a crosslinking process with glutaraldehyde (GA), and L4: electrodeposition of Pt nanoparticles.

#### 2.2.2. Multilayered Membrane Characterization

To study the chemical composition of the membranes, attenuated total reflectance–Fourier transform infrared (ATR-FTIR) measurements were conducted using a Bruker Alpha Platinum-ATR spectrometer (Billerica, MA, USA) in the transmittance mode. Briefly, a membrane coupon was analyzed with a spectral width ranging from 4000 to 500 cm^−1^, with a 4 cm^−1^ resolution, and an accumulation of 64 scans. To characterize the membrane morphology, scanning electron microscopy (SEM) images were taken with a Hitachi S-4800 field emission SEM (Chiyoda-ku, Tokyo, Japan) with an accelerating voltage of 10.0 KV and a current of 5 µA. Samples were air-dried, attached to a sample holder, and sputtered with a gold film (ca. 10 nm thick). Also, an energy-dispersive X-ray spectroscopy (EDS) detector was used (i.e., SEM-EDS) to study the membrane surface composition. The surface wettability of the membranes was analyzed via water contact angle measurement; a Krüss drop shape analyzer DSA25S (Hamburg, Germany) was used at room temperature. A 1 cm^2^ coupon of each membrane was fixed over a glass slide with carbon tape. The analysis started when a 4.50 µL npw droplet was released onto the membrane surface; the data was recorded every 0.5 s up to 60 s and analyzed in real-time using advanced software (version 1.8).

The electrochemical properties of the membrane were evaluated using cyclic voltammetry in the same custom-made 3-electrode setup: (i) in 5 mM Ferri/Ferrocyanide in 0.1 M KCl from −1.2 V to 1.6V vs. Ag/AgCl at 50 mV for 5 cycles, and (ii) in 0.5 M H_2_SO_4_ solution (before and after the Pt deposition) from −0.6 V to 1.4 V vs. Ag/AgCl, at 50 mV/s scan rate for 5 cycles. Electrochemical impedance spectroscopy (EIS) was conducted in 0.5 M H_2_SO_4_ solution; E_oc_ = 0 V, voltage amplitude: 20 mV, frequency range: 7 MHz to 400 Hz and 20 points per decade. The membrane was used as WE (area of 0.13 cm^2^), Ag/AgCl as RE, and Pt wire as CE. All measurements were initialized at the open circuit voltage (OCV), after allowing the system to stabilize for 5 min. Once the OCV stabilized, the scans were initiated, and all analyses were performed relative to the OCV.

The diffusion coefficient of the Ferri/Ferrocyanide redox probe when the different membranes were used as WE was determined with the Randles–Sevcik Equation (1) via CV at different scan rates (5, 10, 20, 30, and 50 mV/s).

(1) $$i_{p}=2.687\times 10^{5}\;n^{3/2}AD^{1/2}C^{0}\nu^{1/2}$$

where *i_p_* is the current peak (e.g., anodic or cathodic), *n* is the number of electrons in the half-reaction of the redox probe (*n* = 1), *A* is the electrode’s geometric area, *D* is the diffusion coefficient, *C* is the bulk concentration, and *ν* is the scan rate.

The FO permeability evaluation was assessed using an acrylic custom-made cross-flow cell with npw as the feed solution (FS) and an aqueous 5% *w*/*v* NaCl solution as the draw solution (DS). The membrane active area was 4.25 cm^2^ and the active layer was facing the FS. The data was collected for 1 h at constant flow rate (12.3 mL/min) and temperature (22 ± 1 °C).

The water fluxes, *J_w_* in LMH (L·m^−2^·h^−1^), were calculated using the following Equation (2):

(2) $$J_{w}=\frac{∆V}{A_{M}t}$$

where Δ*V* is the volume increment in the draw solution in *L*, *A_M_* is the membrane area (m^2^), and *t* is the time of the test in hours.

The reverse salt flux, *J_s_* in GMH (g·m^−2^·h^−1^), from the draw solution to the feed solution was calculated using Equation (3):

(3) $$J_{s}=\frac{∆(C_{t}V_{t})}{A_{M}t}$$

where *C_t_* and *V_t_* are the salt concentration and the feed volume at the end of the FO experiments, respectively. To determine the salt concentration in the feed solution, we monitored the solution conductivity and used a calibration curve of NaCl solutions (i.e., concentration-conductivity).

The ammonia electro-oxidation reaction was studied via CV in a 0.5 M NH_4_OH solution in 1 M KOH (pH > 10). The ammonia-free control was obtained by analyzing the 1 M KOH solution (pH > 10). The studied potential window was from −0.8 V to 0.2 V vs. Ag/AgCl, at a 50 mV/s scan rate for 5 cycles. The membrane was used as WE (area of 0.13 cm^2^), Ag/AgCl as RE, and Pt wire as CE. The AOR was also monitored via chronoamperometry at different NH_4_OH concentrations: 0.250 M, 0.125 M, and 0.0625 M with variations in the applied voltage from −0.1 to −0.5 V vs. Ag/AgCl.

## 3. Results and Discussion

### 3.1. Selection of the Carbon Black as Additive

The composition and intrinsic properties of the support layer can govern the membrane performance. Moreover, interactions between the polymer matrix and the additives used can alter properties like hydrophilicity, solubility, and precipitation rate of the film; the porosity, pore distribution, and pore morphology can be influenced. Here, we embedded porous carbon additives in a similar way to the reported approaches [30,31]. Carbon black (CB) particles were chosen owing to their porosity, surface area, and good interaction with polymers [32]. We studied three types of CBs: (a) black pearls 430 (BP4), (b) black pearls 280 (BP2), and (c) Vulcan XC-72 (VXC). The main difference among these materials is their particle size, oil absorption number (OAN), densities, and porosity (comparison in Table S1). These aspects define how the CB particles can interact with polymer-based materials, and therefore, the overall functionality of the fabricated membrane.

We analyzed the untreated CBs using the Brunauer–Emmett–Teller (BET) method to determine the specific surface area of the material. After completing the measurement (results in Table S1), we determined that VXC has the highest surface area (221.89 m^2^/g), followed by BP4 (77.04 m^2^/g), and the lowest was BP2 (42.20 m^2^/g). These results showed a significant difference in surface area among the studied CB particles which can be correlated to their particle size and intrinsic porosity. Then, we embedded these CBs within the polysulfone support by adding them into the casting solution. We tested the FO performance of the carbon black embedded supports (CBES) using npw as the feed solution and NaCl as the draw solution. In this case, as shown in Table S1, the trend was BP4 (37.4 ± 14 LMH) > VXC (20.2 ± 4 LMH) > BP2 (14.2 ± 11 LMH). We suggest that the significant difference in water flux is due to the inherent properties of the BP4. Specifically, BP4 has larger particles and the highest density, which implies it could interact better with the PSF chains within the casting solution. It also has the lowest OAN, so fewer molecules of the solvent in the casting solution can be absorbed, and a faster precipitation rate in the phase inversion method occurs. All these properties translate into higher porosity within the support layer and higher water flux. For this reason, we selected the BP4 as the optimal additive to incorporate into the membrane support. Thus, we will be referring to the support with BP4 as the additive when mentioning CBES for the rest of this work.

On the other hand, the deposition of additives in the membrane active layer (i.e., surface) can attribute specific features to it [20,33]. Herein, we were interested in an electroactive layer capable of extending the electrochemical features to the surface while acting as a substrate for the Pt electrocatalyst. VXC has previously been used for this task [23,28,34] and here, showed the smallest particle size, exhibits the highest surface area, and also has the highest OAN (i.e., ability to absorb liquids). In light of this, VXC was selected as the surface additive in the membrane layout.

### 3.2. Carbon Black Embedded Support (CBES)

**Figure 1:**
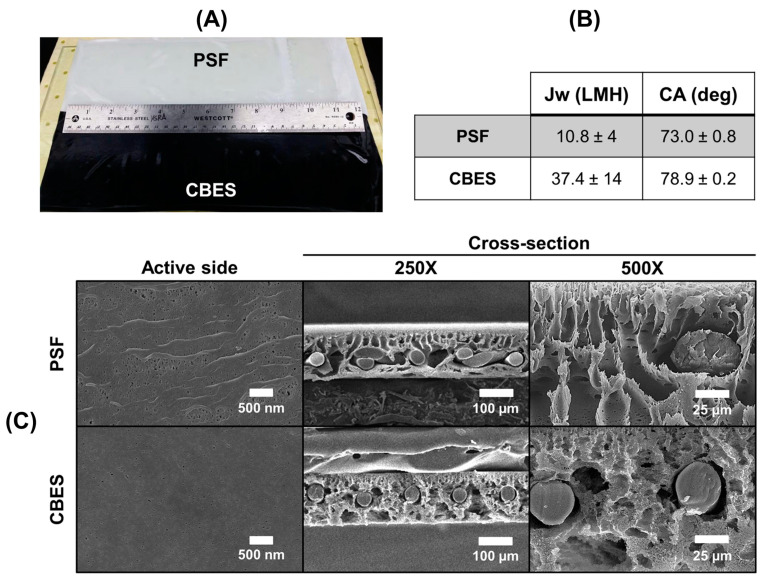
Physical comparison between the PSF and CBES supports. (**A**) Photograph of the fabricated supports, (**B**) summary of the water flux (*J_w_*) and contact angle (CA), and (**C**) SEM micrographs of the surface and cross-section.

Two polymeric supports were fabricated to study the effect of CB particles on the membrane performance (see Figure 1A): (A) the control made of polysulfone (PSF) and (B) the CBES by mixing PSF and BP4. PSF is a polymer that has been thoroughly studied as the support layer of TFC membranes [35]. The incorporation of carbonaceous species results in a practical carbon-embedded support due to the high chemical inertness and electrical conductivity of the CB particles [36,37]. It has also been shown that the incorporation of non-polymeric substances into polymeric matrixes can enhance the membrane flux while avoiding both membrane swelling and compromising the mechanical stability of the membrane [38].

We determined that BP4 was the best CB additive to incorporate into the polymeric matrix of the support because it reached a higher water flux in the FO experiment. To understand the rationale of this performance, we characterized and compared the CBES with the PSF support. First, we analyzed the chemical composition using ATR-FTIR. The FTIR spectra of PSF in Figure S1A show its characteristic peaks corresponding to its chemical skeletal vibrations [39]. These vibrations include the aromatic ring stretching, the SO_2_ antisymmetric and symmetric stretching, scissoring, and the C–O–C and C–S stretching (vibrations summarized in Table S2). These PSF characteristic peaks were present in the CBES support, indicating there were no chemical interactions between the PSF chains and the CB particles. Also, the C–H stretching in aromatics and alkyl is present in both supports. In Figure S1B, the CBES FTIR spectra show the distinct O–H stretching band at 3288 cm^−1^ confirming the incorporation of additional functional groups characteristic of CB particles. Also, an increase in intensity in the CO and CO_2_ adsorption regions is noticeable, region 2490–2160 cm^−1^, and peak at 2087 cm^−1^, respectively, suggesting that the incorporated BP4 particles increase the membrane adsorption capacity. These FTIR spectra confirmed the chemical changes by incorporating CB particles into the polymeric support.

Then, we analyzed the wettability of each support by using water contact angle measurements of the surface. The PSF support had an angle of 73.0 ± 0.8° while the CBES was 78.9 ± 0.2°. This result showed that incorporating BP4 into the support slightly increases the hydrophobic character of the polymeric support. The predominant sp^2^ carbons in CB particles can be the main reason for increasing the water contact angle of the support surface. Yet, the water flux of the bare PSF was 10.8 ± 4 LMH while the CBES surpasses this performance by a 4-fold increment. This substantial increment in water flux can be attributed to the porosity. As shown in Figure 1C, especially in the SEM cross-section images, the addition of BP4 into the polymeric support enhances the membrane porosity. On the surface, the PSF has wave-like textures and larger pores while the CBES support has a flatter surface and smaller pores (see also Figure S2). Notable differences are spotted in the cross-section images. Within the PSF support, the pores have a finger-like structure and are more uniformly distributed along the film. In contrast, the pores in the CBES have a mixed morphology between finger-like and sponge-like structures in addition to having seemingly higher porosity (i.e., with smaller pores). These results are in agreement that the CB particles affect the membrane pores morphology, distribution, and size which resulted in an enhanced support with higher water permeability.

**Figure 2:**
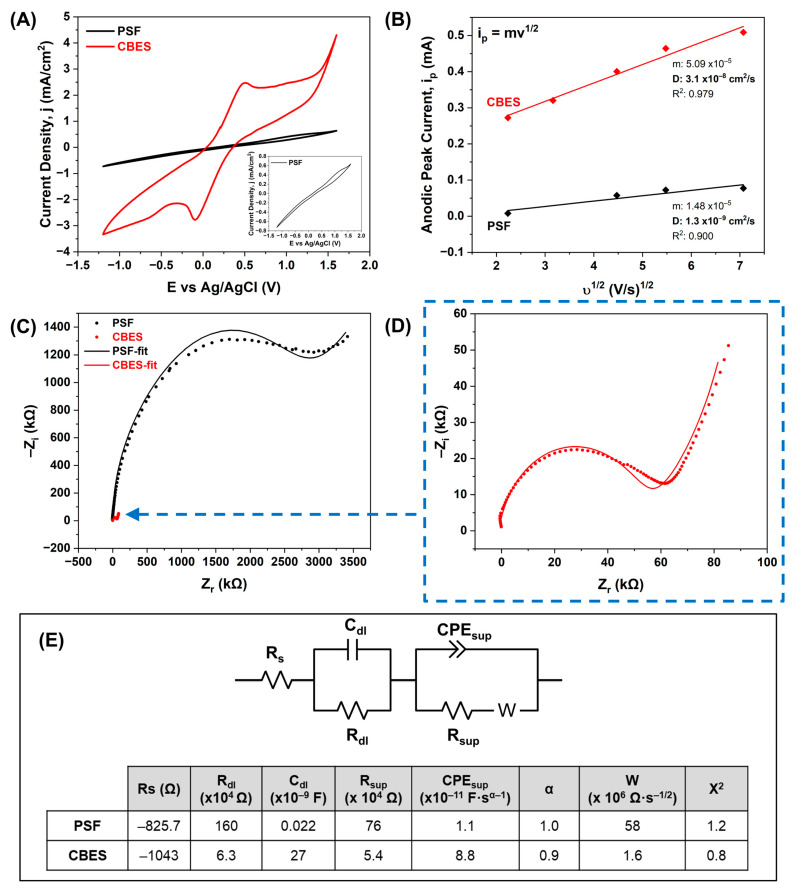
Electrochemical evaluation of the PSF and CBES supports in a three-electrode cell where the supports were used as working electro (WE), Pt wire was used as a counter electrode (CE), and Ag/AgCl (0.197 V vs. NHE) as the reference electrode (RE). (**A**) Cyclic voltammograms using the ferri/ferrocyanide redox couple (5 mM in 0.1 M KCl) at 50mV/s, (**B**) Randles–Sevcik plot to determine the ferri/ferro diffusion coefficient, (**C**) Nyquist plot to compare the membrane impedance, (**D**) CBES Nyquist plot close-up, and (**E**) Randles EEC and the respective values of each electrical component.

We proceeded to evaluate the effect of adding conductive carbonaceous particles onto the polymeric support in terms of electroactivity. To do so, we completed cyclic voltammetry (CV) measurements using the ferri/ferrocyanide redox couple. The voltammogram in Figure 2A showed meaningful differences between the PSF and CBES. The minimal electrochemical response on PSF can be attributed to the permeation of the redox couple through the porous PSF membrane. Furthermore, on the CBES membrane, the one-electron redox transfer of the ferri/ferrocyanide redox couple is present which corroborates that the embedded BP4 particles, even within a non-conductive polymer matrix are capable of promoting an electrical response. To further understand this enhancement in electrical response, we studied the ferri/ferrocyanide redox reaction as a function of scan rate (Figure S3). Faster scan rates led to higher currents owing to a decrease in the diffusion layer at the membrane surface. The CBES voltammogram shows the reversible redox reaction of the redox couple. To understand the kinetics of this redox reaction, the Randles–Sevcik equation (Equation (2)) was used, and the resulting linear behaviors are shown in Figure 2B. The CBES has a higher slope at the *i_p_-v*^1/2^ plot and diffusion coefficient (3.1 × 10^−8^ cm^2^/s) meaning that the redox probe has a faster diffusion through the support.

Thereafter, we evaluated the electrochemical impedance (EIS) of the supports. Figure 2C shows the comparison of the Nyquist plot, where a significant reduction of the overall impedance was achieved by the CB. Figure 2D shows a closer examination of the CBES Nyquist plot. In an attempt to provide an insight into the electrical response attributed to the embedded CB particles, we determined the Randles equivalent electrical circuit (EEC). The EEC that fitted the results is represented in Figure 2E and the respective values for each component account for the effect of each layer in the membrane. The EEC is composed of the bulk or solution resistance (R_s_), i.e., ohmic resistance, and is present at higher frequencies. Both supports have negative R_s_, this trend has been previously reported for complex systems when the electrolytes passivate the studied surface [40].

The next RC circuit corresponds to the ionic double-layer formed at the membrane surface. The incorporation of the CB particles in the polymeric support reduces the R_dl_ by a 100× factor (PSF: 160 × 10^4^ Ω and CBES: 6.3 × 10^4^ Ω) due to a significant reduction of the ionic double layer. A smaller double layer can be attributed to a more defined arrangement. The CBES incorporates the CB particles’ functional groups which can dictate the arrangement of the ion charges on the surface whilst in the PSF an uncontrolled double layer is formed. The double-layer capacitance (C_dl_) is increased by a 1000× factor when the CB particles are embedded into the polymeric support (PSF: 22 pF and CBES: 26 nF), which corroborates that the CBES surface has an improved charge conductivity/mobility.

Lastly, in the circuit that describes the support inner pores, the same trend was observed where the embedded CB particles reduced the resistance (R_sup_; from 76 × 10^4^ Ω to 5.4 × 10^4^ Ω) while also increasing the capacitance represented by the constant phase element (CPE). Thus, the internal membrane resistance was reduced which can be attributed to the increment in porosity which can accommodate a higher number of electrolytes and increase the effective energy storage of the support. The linear section at lower frequencies on the Nyquist plot corresponds to a Warburg element (W) which has been associated with diffusion; in this matter, it can be related to the membrane inner pores and their capacity to diffuse the electrolytes.

Generally, the CB embedded in the support provides considerable changes to the overall physical (e.g., color and porosity), morphological (e.g., pores size and arrangement), and electrical properties of the membrane. Moreover, higher porosity, rearranged pore morphology, and smaller pores were attained. Regarding the electrical properties, the impedance was significantly reduced and suitable diffusion properties and enhanced capacitance were achieved. To test the support ability to conduct a more complex redox reaction, we tried the AOR reaction. Figure S4 confirmed that the electrochemical properties of the CBES are not enough to complete the AOR. Nonetheless, the results presented here demonstrate that although CB particles are non-refined carbonaceous species and do not exhibit a controlled structure like graphene or CNTs, they can be considered an abundant and inexpensive additive in the membrane design.

### 3.3. Hydrophilic and Electroconductive Active Layer

**Figure 3:**
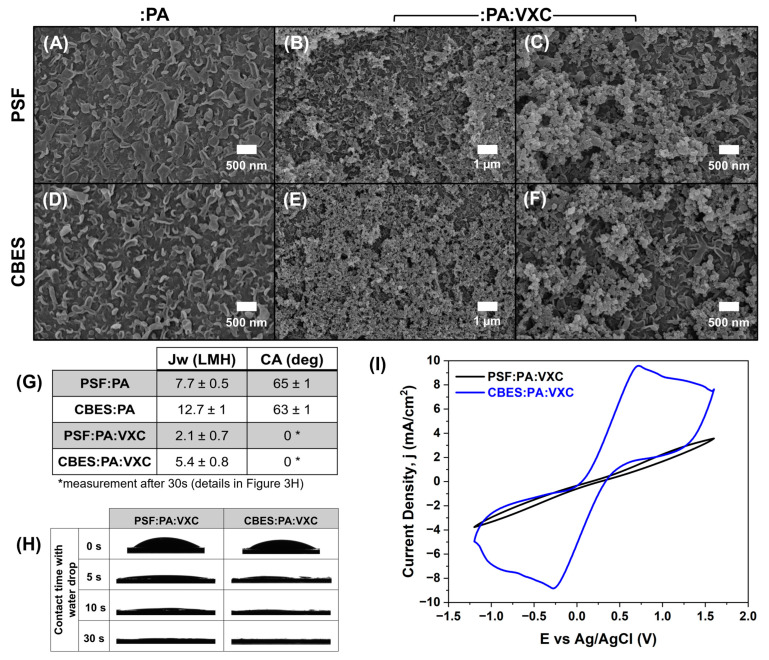
Evaluation of the :PA and :VXC layers over the PSF and CBES supports. (**A**–**F**) Morphological comparison via SEM images, (**G**) summary of water flux (*J_w_*) and contact angle (CA), (**H**) continuous water contact angle results of the membranes containing the :PA and :VXC layers, and (**I**) electrochemical evaluation via cyclic voltammetry (CV) using ferri/ferrocyanide redox couple (5 mM in 0.1 M KCl, RE: Ag/AgCl (NaCl saturated, 0.197V vs. NHE) and CE: Pt wire, and a scan rate of 50 mV/s).

To increase the membrane hydrophilicity and selectivity while preventing the pores from clogging after the VXC layer deposition, the support’s surface was modified with a thin layer of polyamide (PA) through interfacial polymerization. The PA layer has been widely used in thin film composite (TFC) membranes due to their stability and highly crosslinked matrix [41]. The polymerized PA exhibited the characteristic ridge and valley morphology; it has been reported that this morphology (showed in Figure 3A,D) of the PA accounts for good water permeability [42]. The membrane’s surface exhibited lower contact angles than that of the bare supports, PSF:PA 65 ± 1° (8% reduction), and CBES:PA: 63 ± 1° (16% reduction) due to the addition of the PA hydrophilic functional groups.

Thereafter, the VXC particles were spray-coated over the X:PA (where X: PSF or CBES) membrane. The immobilization of the VXC over the PA layer was promoted by a crosslinking between the spray-coated PVA and GA in acidic conditions [43]. The surface morphology of the 3-layer membranes (X:PA:VXC) was studied via SEM. As shown in Figure 3B,E, the crosslinked VXC layer shows a rough granular surface which is often associated with higher porosity and surface area. After a closer examination in higher magnification SEM images (Figure 3C,F), the PA layer can be observed at the bottom of the VXC (see also Figure S5). The VXC layer created a superhydrophilic surface where the contact angle was 0° in less than 60s of contact with the npw drop (Figure 3H). It has been reported that, typically, CBs are hydrophobic [44]. Nonetheless, as reported by Han-Ying, L. et al. and Li, J. et al. the CB particles can be encapsulated by a polymer [45,46]. In our study, PVA and GA were crosslinked to immobilize the VXC over the membrane surface but also accounted for the change in hydrophilicity of the CBs due to their richness in -OH [43] functionalities. As reported in Figure 3G, the membrane porosity was also enhanced by 10% when compared to X:PA. These results showed that the physico-chemical properties of the membrane are not affected by the chemical composition of the support layer.

On the other hand, the electrical response of the membrane is influenced by the composition of the polymeric support. We studied the electrochemical properties of the membranes via a CV using the ferri/ferrocyanide redox couple. The voltammogram in Figure 3I shows that the electron transfer of the redox couple is more suitable with the VXC layer over the CBES:PA than over the PSF:PA. Therefore, the CB particles embedded in the support intensify the membrane electrochemical capabilities. If we compare these voltammograms with the ones in Figure 2A, we noticed that in the VXC layer over PSF:PA increases the non-faradaic current while a more defined one-electron transfer reaction is shown for the CBES:PA:VXC; almost a 3-fold increase was reached in both anodic and cathodic currents. These results suggest that the CB particles in the support layer and the active layer can work synergistically and stimulate an interconnected electroconductive matrix. To evaluate the VXC electrocatalytic properties, the AOR was studied. The voltammograms in Figure S6 showed that there is no charge transfer reaction validating the need for a specific electrocatalyst towards the ammonia oxidation.

**Figure 4:**
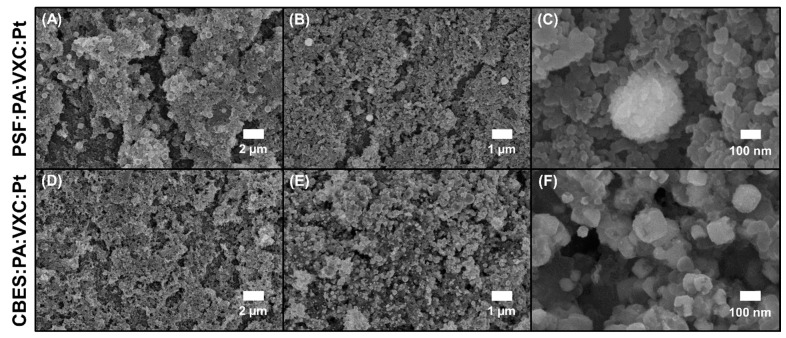
Surface morphology comparison at different SEM magnifications (5k, 10k, and 100k) between (**A**–**C**) PSF:PA:VXC:Pt and (**D**–**F**) CBES:PA:VXC:Pt.

To enhance the membrane catalytic properties towards the AOR, we electrodeposited platinum nanoparticles (PtNPs) over the VXC layer of the membranes. PtNPs were selected as the electrocatalyst to provide selective sites where the ammonia electro-oxidation can be completed [47]. The presence of the PtNP was corroborated physically with SEM and chemically via EDS. As shown in Figure 4, both membrane surfaces (PSF- and CBES-based) exhibited new spherical-like particles. Homogeneous deposition can be corroborated at smaller magnifications (Figure 4C,F). The particles over the PSF-based support have an average size of 350 nm, while they are smaller over the CBES-based support, with an average size of 50–200 nm. The variation in particle size can be attributed to the overall membrane electro-responsiveness, and diffusion ability to electrochemically reduce platinum salt. The SEM-EDS was used to analyze the chemical composition of some of the particles (Figure S7). These results confirmed that the spherical-like particles are composed of Pt^0^. Thus, the PtNPs are well-distributed and exhibit continuity along the surface, which allows for a better resolution of the electrochemical profile. The PtNPs are over the cross-linked VXC-PVA-GA, thus, less -OH functional groups can be exposed which results in a decrease in membrane hydrophilicity. The slight increase in the surface contact angle is: 15 ± 1° for PSF:PA:VXC:Pt and 19.8 ± 0.4° for CBES:PA:VXC:Pt.

**Figure 5:**
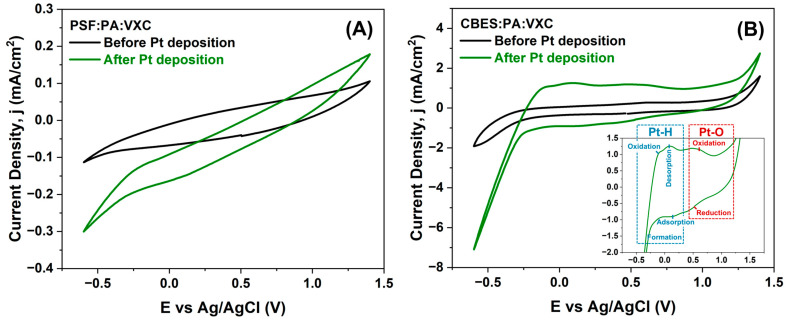
Cyclic voltammogram of (**A**) PSF:PA:VXC and (**B**) CBES:PA:VXC before and after the Pt electrodeposition at a scan rate of 50 mV/s in 0.5 M H_2_SO_4_. The RE was Ag/AgCl (NaCl saturated, 0.197 V vs. NHE) and the CE was a Pt wire. The inset graph in (**B**) corresponds to a higher magnification of the Pt electrochemical processes.

To determine if the PtNPs are electrochemically active, we performed CV in acid media (Figure 5A,B) under the same experimental conditions as the electrodeposition. In Figure 5A, the voltammogram of the PSF:PA:VXC:Pt membrane, the Pt deposition did not affect the non-faradaic current but higher cathodic and anodic currents were reached. Also, the increment in the cathodic current at −0.6 V vs. Ag/AgCl corresponds to the hydrogen evolution reaction (HER) triggered by the PtNPs. In the voltammograms in Figure 5B, the differences before and after the Pt deposition are remarkable. The CBES:PA:VXC:Pt membrane exhibits the characteristic electrochemical behavior of Pt in acidic conditions [48]. A cathodic current associated with the hydrogen evolution reaction (HER) at −0.2 V vs. Ag/AgCl is observed; in this step, the hydrogen is adsorbed and then Pt-H species are formed. In the anodic sweep, hydrogen is oxidized and desorbed from the PtNPs, and its metallic nature is regained until 0.6 V vs. Ag/AgCl where the Pt-oxides are formed. These oxides are subsequently reduced to Pt[0] in the cathodic sweep at 0.5 V vs. Ag/AgCl. As a result, the PtNPs were electrochemically validated in the CBES:PA:VXC:Pt membrane. Moreover, the responsiveness of the membrane in acidic conditions confirms that the PtNPs are electrochemically active and behave similarly to other Pt catalysts [49,50].

**Figure 6:**
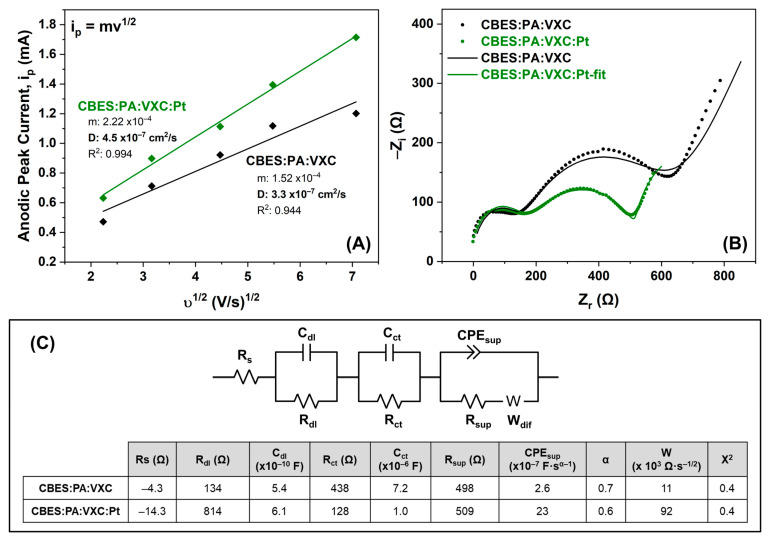
Electrochemical evaluation and comparison between CBES:PA:VXC and CBES:PA:VXC:Pt. (**A**) Randles–Sevcik plot to determine the diffusion coefficient, (**B**) Nyquist plot to study the membrane’s impedance, and (**C**) Randles EEC and estimated values for each electrical component.

Additional electrochemical measurements were performed to understand how the PtNPs affect the overall electrical response. The results from Figure S8 were interpreted for the Randles–Sevcik plot shown in Figure 6A as a comparison between the :VXC and :VXC:Pt over the CBES. The slope and the diffusion coefficient are higher in the presence of the PtNPs indicating an enhancement in the charge-transfer redox reaction. EIS was also used to study the interfacial properties when the VXC and the PtNP we added, respectively (Figure 6B). The Randles EEC of the membranes (shown in Figure 6C) has an additional RC circuit than the PSF and CBES support due to the incorporation of the conductive material. Similar to the polymeric supports, the R_s_ are also negative. The R_dl_ was increased after the PtNP’s were electrodeposited from 134.4 Ω to 814.3 Ω. This is attributed to the variation in roughness and intrinsic charge of the membrane surface. Therefore, the VXC:Pt layer can accommodate more electrolyte species in the double layer, yet it has a higher capacitance. The additional RC circuit corresponds to the charge-transfer process that occurs at the electroactive material, VXC and VXC:Pt. The R_ct_ is smaller in the presence of the Pt nanoparticles suggesting a faster electron transfer of the electrolyte, which is consistent with the diffusion coefficient trend. Lastly, the C_sup_ (i.e., CPE) is also increased by a 10× factor in the membrane with all the deposited layers (CBES:PA:VXC:Pt) which validates the synergistic effect among the layers to increase the overall membrane ability to hold charged species.

These results confirmed that the fabricated multilayered active layer was hydrophilic and electroactive. Moreover, the VXC particles proved to be a suitable substrate for the electrodeposition of PtNPs on a membrane surface and enhanced the charge-transfer kinetics of different redox reactions. Additionally, the CBs at the different layers altogether significantly reduced the inner pores resistance allowing good electrical mobility and increasing the effective reaction area and energy storage capacity.

### 3.4. Multilayered Active Layer Performance Evaluation

**Figure 7:**
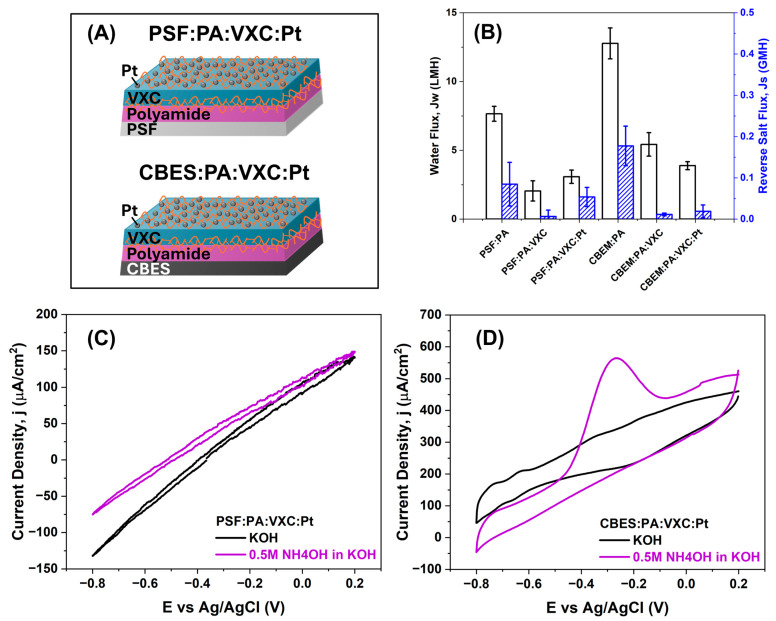
Carbon black embedded support and membrane. (**A**) Schematic representation of all the layers in the fabricated membrane, (**B**) FO performance comparison including the water flux (*J_w_*) and reverse salt flux (*J_s_*), (**C**) AOR evaluation using PSF:PA:VXC:Pt as the WE, and (**D**) AOR evaluation using CBES:PA:VXC:Pt as the WE.

After the fabrication of all the membranes, we evaluated their performance in terms of their capacity in FO filtration and electrochemical ammonia electro-oxidation, respectively. First, we studied the FO membranes [51]. Here, we fabricated two four-layer membranes (represented in Figure 7A), and it can be observed that the addition of each layer of the composite membrane brings about a reduction in water flux (*J_w_)*, and consequently, in reverse salt flux (*J_s_*). The control membranes (PSF and CBES) are represented in Figure S9, where CBES has the highest *J_w_* performance in a custom-made cell, using npw water as the feed solution and 5% *w*/*v* NaCl as the draw solution. The results shown in Figure 7B follow trends in previous studies of multilayered and large *J_s_*. The addition of the PA layer, which created a dense cross-linked layer, significantly reduces the water flux as well as the reverse flux when compared to the pristine supports. Nevertheless, the reduction in both fluxes (*J_w_* and *J_s_*) with the additional VXC layer is less significant than that with the PA layer, and the PtNPs do not seem to alter the permeability due to their superhydrophilic character. It should be noted that even though the water permeability was reduced significantly by the hydrophilic-electroactive layers, the multilayered membranes were still able to selectively permeate water with *J_w_* of 3.1 ± 0.5 LMH (PSF:PA:VXC:Pt) and 3.9 ± 0.3 LMH (CBES:PA:VXC:Pt).

As a proof-of-concept, we evaluated the multilayer membrane capacity to complete the electro-oxidation of ammonia. Similar to J.-H.K. et al., we explored its potential by using higher concentrations than typical wastewater conditions; this allowed a comprehensive study on the diffusion-controlled process [52]. The electrocatalytic activity of the multilayered membranes for AOR was investigated in an alkaline aqueous solution; KOH was used as the electrolyte. The membranes were employed as WE, and via CV the electrochemical reaction was monitored. The membranes without PtNPs were evaluated and no redox reactions occurred. The voltammograms for the PSF:PA:VXC:Pt membrane are shown in Figure 7C where no significant differences are observed in ammonia-free solution or the presence of ammonia. On the contrary, the CBES:PA:VXC:Pt membrane (Figure 7D) in the presence of ammonia showed an oxidation peak at −0.3 V vs. Ag/AgCl which corresponds to the AOR [10,11,53]. It is known that Pt is an excellent electrocatalyst for the AOR. However, polymeric membranes are non-conductive, thus, these results demonstrated that the membrane construction provided a suitable interface where the charge- transfer was accomplished. Moreover, the successful ammonia oxidation confirmed that integrating different materials in a layered approach allowed an electrical continuation throughout the membrane polymeric support. This can be sustained by the fact that the PSF:PA:VXC:Pt membrane had the same surface modifications, including the presence of the Pt electrocatalyst yet was unable to complete the AOR. This suggests that the polymeric support’s resistance is a critical parameter that needs to be reduced, and the embedded CB particles proved to be effective.

**Figure 8:**
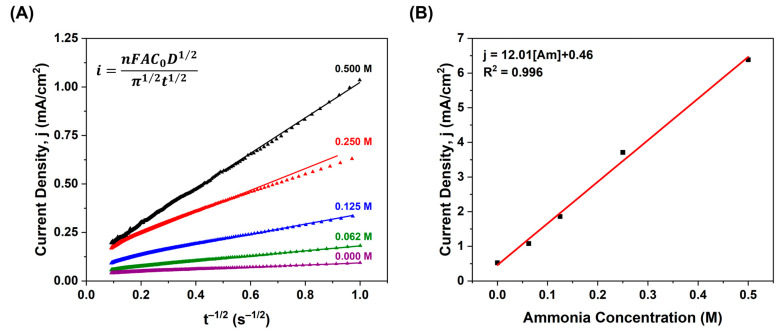
Evaluation of the AOR activity over the CBES:PA:VXC:Pt membrane at different ammonia concentrations. (**A**) *j-t^−^*^1/2^ plot at constant −0.3 V vs. Ag/AgCl and (**B**) *j-c* plot at −0.3 V vs. Ag/AgCl.

To further investigate the membrane’s AOR activity, we monitored the reaction at constant potentials (−0.1, −0.2, −0.3, −0.4, and −0.5 V vs. Ag/Cl) for 120 s. These chronoamperometry experiments were carried out to examine the activity and stability of the membrane. Figure S10A shows that the maximum current was obtained at −0.3 V vs. Ag/AgCl, the oxidation potential determined in the CV. Also, we completed this study by varying the ammonia concentration (0.250 M, 0.125 M, and 0.0625 M) and noted a concentration dependence. The same current decay was observed but, in this case, the maximum current was reduced proportionally to the concentration. From these results, we used the Cottrell equation shown in Figure 8A and plotted the *j-t^−1^*^/*2*^ as shown in Figure S10B. We conducted the same Cottrell analysis at a constant −0.3 V vs. Ag/AgCl and monitored the changes attributed to variations in ammonia concentration (Figure 8A); the comparative chronoamperometry is shown in Figure S11. In the chronoamperometry and Cottrell analyses, the same current decay was followed. These results suggest that the AOR reaction over the fabricated membrane is a diffusion-controlled redox process. Additionally, we plotted *j* vs. concentration, as shown in Figure 8B, and found there is a linear relationship between the ammonia concentration and the generated current at −0.3 V vs. Ag/AgCl. This confirms the diffusion-controlled process but also is indicative that the main reaction occurring at this potential is the ammonia oxidation, and no other secondary processes such as adsorption are evident.

## 4. Conclusions

In this work, we developed a multilayered membrane with a hydrophilic and electrocatalytic surface. This membrane was composed of carbon black particles which are an uncommon carbonaceous material used in FO membrane-based technologies. Even though the CB particles are a non-refined material, they exhibit high surface area and when embedded within a polymeric support, they can alter the pore morphology which caused an increase in membrane porosity and in water permeability. Moreover, the CBES exhibited electrochemical features; the impedance of the membrane was significantly reduced which led to smaller resistance within the inner pores of the polymeric support. Furthermore, the additional layers on the membrane surface: PA:VXC demonstrated to work synergistically with the support material. The membranes with the CBES support have a smaller double-layer and support resistance when compared to the unmodified PSF support. We systematically compared the effect of the addition of each layer material and the results showed that the physico-chemical properties of the active layer were very similar in both PSF- and CBES-based membranes. Thus, the main differences in electrochemical response are strictly attributed to the development of a cohesive electrical mobility throughout the layers.

This work proved that the VXC particles are a good substrate over a porous membrane where the PtNP can be electrodeposited. Furthermore, the PtNP was validated as physically, chemically, and electrochemically active. The reduction in the overall membrane impedance was significant and an additional charge-transfer process was confirmed with EIS. Overall, the CBES:PA:VXC:Pt membrane outperformed the PSF-based membrane in all the electrochemical evaluations. Most importantly, we demonstrated a proof-of-concept where this multilayered membrane completed the AOR at −0.3 V vs. Ag/AgCl. The activity was validated at different applied potentials and ammonia concentrations. The linear responsiveness in chronoamperometry and Cottrell plots confirmed a diffusion-controlled process, and no secondary processes were apparent. Our findings demonstrated that for the first time, a FO multilayered membrane with CB particles as the electrochemical additive can exhibit hydrophilicity, electrocatalytic, water permeability, and selectivity, and potentially can be employed to remove ammonia from contaminated water sources. Moreover, the negative applied potential to conduct the AOR could hypothetically charge the membrane surface negatively which could simultaneously prevent membrane fouling by another negatively charged foulants.

In general, both properties, the water permeability and AOR properties of the membrane were validated separately. In future studies, we will evaluate the filtration-AOR in situ with a custom-made FO setup. For these experiments, synthetic wastewater will be explored, and the ammonia concentration will be monitored to quantify the degradation while studying any possible intermediates during the oxidation process. Lastly, this will allow us a more comprehensive energetical analysis of the process for wastewater.

## Data Availability

The raw data supporting the conclusions of this article will be made available by the authors on request.

## Supplementary Materials

The following supporting information can be downloaded at: https://www.mdpi.com/article/10.3390/membranes15020037/s1. Supporting information contains: Table S1: Comparison of the CB reported properties1, and the experimental results of the BET surface area and water flux; Figure S1. ATR-FTIR spectra of the fabricated supports (A) PSF and (B) CBES; Table S2. Summary of the peak location of the infrared bands of the main chemical functional groups in the fabricated supports: PSF and CBES; Figure S2. SEM micrograph at a 100,000× magnification; Figure S3. CV of Ferro/Ferricyanide at different scan rates (mV/s) using (A) PSF and (B) CBES as the working electrode; Figure S4. Ammonia electro-oxidation evaluation at different scan rates using CBES as the working electrode. Cyclic voltammograms were performed using 1M KOH as electrolyte. A Pt wire was used as counter electrode (CE) and Ag/AgCl (0.197V vs. NHE) as the reference electrode (RE); Figure S5. SEM micrographs at higher magnifications of the active layers: PA and PA:VXC over PSF and CBES; Figure S6. Ammonia electro-oxidation evaluation of (A) PSF:PA:VXC and (B) CBES:PA:VXC. Cyclic voltammograms were performed using 1M KOH as electrolyte. A Pt wire was used as counter electrode (CE) and Ag/AgCl (0.197V vs. NHE) as the reference electrode (RE); Figure S7. EDS results of both multilayered membranes: PSF:PA:VXC:Pt and CBES:PA:VXC:Pt; Figure S8. CV of Ferro/Ferricyanide at different scan rates (mV/s) using (A) CBES:PA:VXC and (B) CBES:PA:VXC:Pt as the working electrode; Figure S9. Water permeability and reverse salt flux comparison of the PSF and CBES; Figure S10. AOR activity measured at different potentials −0.1 V to −0.5 V vs. Ag/AgCl and at different ammonia concentrations (0.250 M, 0.125 M and 0.0625 M). (A) Chronoamperometry measurements and (B) Cottrell plots; Figure S11. Evaluation of the AOR activity at constant −0.3 V vs. Ag/AgCl potential while varying the NH4OH concentration. The material is complementary to the main work [54,55].

## References

[1] Jorgensen T.C., Weatherley L.R. (2003). Ammonia removal from wastewater by ion exchange in the presence of organic contaminants. Water Res..

[2] Almutairi A., Weatherley L.R. (2015). Intensification of ammonia removal from waste water in biologically active zeolitic ion exchange columns. J. Environ. Manag..

[3] Qiang J., Zhou Z., Wang K., Qiu Z., Zhi H., Yuan Y., Zhang Y., Jiang Y., Zhao X., Wang Z. (2020). Coupling ammonia nitrogen adsorption and regeneration unit with a high-load anoxic/aerobic process to achieve rapid and efficient pollutants removal for wastewater treatment. Water Res..

[4] Zhang G., Ruan J., Du T. (2020). Recent Advances on Photocatalytic and Electrochemical Oxidation for Ammonia Treatment from Water/Wastewater. ACS EST Eng..

[5] Rohani R., Yusoff I.I., Khairul Zaman N., Mahmood Ali A., Rusli N.A.B., Tajau R., Basiron S.A. (2021). Ammonia removal from raw water by using adsorptive membrane filtration process. Sep. Purif. Technol..

[6] Li P., Jin L. (2022). Efficient Removal Technology of Ammonia Nitrogen by Membrane Separation. Chem. Eng. Technol..

[7] Jafarinejad S., Park H., Mayton H., Walker S.L., Jiang S.C. (2019). Concentrating ammonium in wastewater by forward osmosis using a surface modified nanofiltration membrane. Environ. Sci. Water Res. Technol..

[8] Lee W., An S., Choi Y. (2021). Ammonia harvesting via membrane gas extraction at moderately alkaline pH: A step toward net-profitable nitrogen recovery from domestic wastewater. Chem. Eng. J..

[9] Siddharth K., Chan Y., Wang L., Shao M. (2018). Ammonia electro-oxidation reaction: Recent development in mechanistic understanding and electrocatalyst design. Curr. Opin. Electrochem..

[10] Pillai H.S., Xin H. (2019). New Insights into Electrochemical Ammonia Oxidation on Pt(100) from First Principles. Ind. Eng. Chem. Res..

[11] Jiang Z., Yu T., Chen J., Tan K., Deng R., Zhou A., Yin S. (2023). Regulating Competitive Adsorption on Pt Nanoparticles by Introducing Pb to Expedite Hydrogen Production via Ammonia Oxidation. ACS Appl. Nano Mater..

[12] Sun M., Wang X., Winter L.R., Zhao Y., Ma W., Hedtke T., Kim J.-H., Elimelech M. (2021). Electrified Membranes for Water Treatment Applications. ACS EST Eng..

[13] Ronen A., Walker S.L., Jassby D. (2016). Electroconductive and electroresponsive membranes for water treatment. Rev. Chem. Eng..

[14] Liu H., Ni X.Y., Huo Z.Y., Peng L., Li G.Q., Wang C., Wu Y.H., Hu H.Y. (2019). Carbon Fiber-Based Flow-Through Electrode System (FES) for Water Disinfection via Direct Oxidation Mechanism with a Sequential Reduction-Oxidation Process. Environ. Sci. Technol..

[15] Straub A.P., Bergsman D.S., Getachew B.A., Leahy L.M., Patil J.J., Ferralis N., Grossman J.C. (2021). Highly Conductive and Permeable Nanocomposite Ultrafiltration Membranes Using Laser-Reduced Graphene Oxide. Nano Lett..

[16] Jung B., Ma S., Miang Khor C., Khalid Khanzada N., Anvari A., Wang X., Im S., Wu J., Rao U., Kyoungjin An A. (2023). Impact of polarity reversal on inorganic scaling on carbon nanotube-based electrically-conducting nanofiltration membranes. Chem. Eng. J..

[17] Ahmed F.E., Hashaikeh R., Hilal N. (2019). Fouling control in reverse osmosis membranes through modification with conductive carbon nanostructures. Desalination.

[18] Gol R.M., Jewrajka S.K. (2014). Facile in situ PEGylation of polyamide thin film composite membranes for improving fouling resistance. J. Membr. Sci..

[19] Nikolaeva D., Langner C., Ghanem A., Rehim M.A., Voit B., Meier-Haack J. (2015). Hydrogel surface modification of reverse osmosis membranes. J. Membr. Sci..

[20] Chen Y., Ge Q. (2019). A Bifunctional Zwitterion That Serves as Both a Membrane Modifier and a Draw Solute for Forward Osmosis Wastewater Treatment. ACS Appl. Mater. Interfaces.

[21] Fan X., Wei G., Quan X. (2023). Carbon nanomaterial-based membranes for water and wastewater treatment under electrochemical assistance. Environ. Sci. Nano.

[22] Mandal M., Secanell M. (2022). Improved polymer electrolyte membrane water electrolyzer performance by using carbon black as a pore former in the anode catalyst layer. J. Power Sources.

[23] Siller-Ceniceros A.A., Sánchez-Castro M.E., Morales-Acosta D., Torres-Lubian J.R., Martínez G E., Rodríguez-Varela F.J. (2017). Innovative functionalization of Vulcan XC-72 with Ru organometallic complex: Significant enhancement in catalytic activity of Pt/C electrocatalyst for the methanol oxidation reaction (MOR). Appl. Catal. B Environ..

[24] Saini D., Gunture, Kaushik J., Aggarwal R., Tripathi K.M., Sonkar S.K. (2021). Carbon Nanomaterials Derived from Black Carbon Soot: A Review of Materials and Applications. ACS Appl. Nano Mater..

[25] Ma J., Tang Y., Lu G., Wang Y., Niu W., Fu D., Zhang K., Bahnemann D.W., Pan J.H. (2023). Incorporating Mesoporous Anatase TiO2 Spheres to Conductive Carbon Black Filled PVDF Membrane for Self-Cleaning Photo(electro)catalytic Filtration. J. Phys. Chem. C.

[26] Xing C., Wang Y., Huang X., Li Y., Li J. (2016). Poly(vinylidene fluoride) Nanocomposites with Simultaneous Organic Nanodomains and Inorganic Nanoparticles. Macromolecules.

[27] Düsenberg B., Tischer F., Seidel A.M., Kopp S.-P., Schmidt J., Roth S., Peukert W., Bück A. (2022). Production and analysis of electrically conductive polymer—Carbon-black composites for powder based Additive Manufacturing. Procedia CIRP.

[28] Xue Q., Huang J.B., Yang D.J., Li B., Zhang C.M. (2021). Enhanced PEMFC durability with graphitized carbon black cathode catalyst supports under accelerated stress testing. RSC Adv..

[29] Cruz-Tato P., Rivera-Fuentes N., Flynn M., Nicolau E. (2019). Anti-Fouling Electroconductive Forward Osmosis Membranes: Electrochemical and Chemical Properties. ACS Appl. Polym. Mater..

[30] Zhao Y., Wang X., Ren Y., Pei D. (2018). Mesh-Embedded Polysulfone/Sulfonated Polysulfone Supported Thin Film Composite Membranes for Forward Osmosis. ACS Appl. Mater. Interfaces.

[31] Duong P.H., Chisca S., Hong P.Y., Cheng H., Nunes S.P., Chung T.S. (2015). Hydroxyl functionalized polytriazole-co-polyoxadiazole as substrates for forward osmosis membranes. ACS Appl. Mater. Interfaces.

[32] Li H., Wu L., Zhang H., Dai W., Hao J., Wu H., Ren F., Liu C. (2020). Self-Assembly of Carbon Black/AAO Templates on Nanoporous Si for Broadband Infrared Absorption. ACS Appl. Mater. Interfaces.

[33] Zirehpour A., Rahimpour A., Arabi Shamsabadi A., Sharifian Gh M., Soroush M. (2017). Mitigation of Thin-Film Composite Membrane Biofouling via Immobilizing Nano-Sized Biocidal Reservoirs in the Membrane Active Layer. Environ. Sci. Technol..

[34] Kaluža L., Larsen M.J., Zdražil M., Gulková D., Vít Z., Šolcová O., Soukup K., Koštejn M., Bonde J.L., Maixnerová L. (2015). Highly loaded carbon black supported Pt catalysts for fuel cells. Catal. Today.

[35] Tan X., Rodrigue D. (2019). A Review on Porous Polymeric Membrane Preparation. Part I: Production Techniques with Polysulfone and Poly (Vinylidene Fluoride). Polymers.

[36] Xu X., Zhang H., Yu M., Wang Y., Gao T., Yang F. (2019). Conductive thin film nanocomposite forward osmosis membrane (TFN-FO) blended with carbon nanoparticles for membrane fouling control. Sci. Total Environ..

[37] Ma J., Zhang C., Yang F., Zhang X., Suss M.E., Huang X., Liang P. (2020). Carbon Black Flow Electrode Enhanced Electrochemical Desalination Using Single-Cycle Operation. Environ. Sci. Technol..

[38] Cho Y.H., Han J., Han S., Guiver M., Park H.B. (2013). Polyamide thin-film composite membranes based on carboxylated polysulfone microporous support membranes for forward osmosis. J. Membr. Sci..

[39] Alosaimi A.M. (2021). Polysulfone Membranes Based Hybrid Nanocomposites for the Adsorptive Removal of Hg(II) Ions. Polymers.

[40] Keddam M., Rakotomavo C., Takenouti H. (1984). Impedance of a porous electrode with an axial gradient of concentration. J. Appl. Electrochem..

[41] Ali Z., Al Sunbul Y., Pacheco F., Ogieglo W., Wang Y., Genduso G., Pinnau I. (2019). Defect-free highly selective polyamide thin-film composite membranes for desalination and boron removal. J. Membr. Sci..

[42] Ridgway H.F., Orbell J., Gray S. (2017). Molecular simulations of polyamide membrane materials used in desalination and water reuse applications: Recent developments and future prospects. J. Membr. Sci..

[43] Rudra R., Kumar V., Kundu P.P. (2015). Acid catalysed cross-linking of poly vinyl alcohol (PVA) by glutaraldehyde: Effect of crosslink density on the characteristics of PVA membranes used in single chambered microbial fuel cells. RSC Adv..

[44] Zakaria N.A., Zaliman S.Q., Leo C.P., Ahmad A.L., Ooi B.S., Poh P.E. (2022). 3D imprinted superhydrophobic polyvinylidene fluoride/carbon black membrane for membrane distillation with electrochemical cleaning evaluation. J. Environ. Chem. Eng..

[45] Li H.-Y., Chen H.-Z., Xu W.-J., Yuan F., Wang J.-R., Wang M. (2005). Polymer-encapsulated hydrophilic carbon black nanoparticles free from aggregation. Colloids Surf. A Physicochem. Eng. Asp..

[46] Li J., Shao X., Zhang B., Wang Z., Ye X., Zhang L., Wang W. (2022). Hydrophilic surface modification of carbon black through a mussel-inspired reaction of tannic acid and diethlyenetriamine. Colloid Polym. Sci..

[47] Wang H., Dekel D.R., Abruña H.D. (2024). Unraveling the Mechanism of Ammonia Electrooxidation by Coupled Differential Electrochemical Mass Spectrometry and Surface-Enhanced Infrared Absorption Spectroscopic Studies. J. Am. Chem. Soc..

[48] Furuya N., Koide S. (1989). Hydrogen adsorption on platinum single-crystal surfaces. Surf. Sci..

[49] Komanicky V., Chang K.C., Menzel A., Markovic N.M., You H., Wang X., Myers D. (2006). Stability and Dissolution of Platinum Surfaces in Perchloric Acid. J. Electrochem. Soc..

[50] Yang L., Li Y., Zou T., Xu F. (2024). Synergetic Effect of SiO_2_ and CeO_2_ as the Noncarbon Composite Support on Significantly Promoting Methanol Oxidation and Oxygen Reduction Reaction. ACS Appl. Eng. Mater..

[51] Liu Y., Zhu J., Zheng J., Gao X., Tian M., Wang X., Xie Y.F., Zhang Y., Volodin A., Van der Bruggen B. (2020). Porous organic polymer embedded thin-film nanocomposite membranes for enhanced nanofiltration performance. J. Membr. Sci..

[52] Kang J.-H., Oh G.-G., Lee B.-J., Im S., Kim W., Kang S., Han J.-H. (2024). Direct Electrooxidation of Ammonia-Enriched Wastewater Using a Bipolar Membrane-Integrated Electrolytic Cell. Water.

[53] Li Z.-F., Wang Y., Botte G.G. (2017). Revisiting the electrochemical oxidation of ammonia on carbon-supported metal nanoparticle catalysts. Electrochim. Acta.

[54] (2012). I. Rubber Compounding Ingredients—Carbon Black—Determination of Oil Absorption Number (OAN) and Oil Absorption Number of Compressed Sample (COAN)..

[55] Ray S.S., Gandhi M., Chen S.-S., Chang H.-M., Dan C.T.N., Le H.Q. (2018). Anti-wetting behaviour of a superhydrophobic octadecyltrimethoxysilane blended PVDF/recycled carbon black composite membrane for enhanced desalination. Environ. Sci. Water Res. Technol..

