# Supplementary material for: Assessing a Multilayered Hydrophilic–Electrocatalytic Forward Osmosis Membrane for Ammonia Electro-Oxidation
Source: Membranes (Basel). 2025 Jan 22;15(2):37. doi: 10.3390/membranes15020037 (PMC11857353; doi:10.3390/membranes15020037)
Supplement: Supplementary file 1 [file membranes-15-00037-s001.zip › membranes-3394720-supplementary.pdf]

SUPPORTING INFORMATION

Assessing a Multilayered Hydrophilic - Electrocatalytic Forward Osmosis Membrane for Ammonia Electro-Oxidation

*Perla Cruz-Tato[1,2], Laura I. Penabad[3], César Lasalde[4], Alondra S. Rodríguez-Rolón[1,2], Eduardo Nicolau[1,2]**

[1] Department of Chemistry, University of Puerto Rico – Río Piedras Campus, 17 University Ave 1701, San Juan, PR 00925, USA

[2] Molecular Sciences Research Center, 1390 Ponce De Leon Ave, Suite 2, San Juan, PR 00931, USA

[3] Department of Chemistry, University of Michigan, 930 N University Ave, Ann Arbor, MI 48109, USA

[4] Department of Applied Physics and Materials Science, California Institute of Technology, 1200 E California Blvd, Pasadena, CA 91125, USA

Corresponding Author

*Phone: 787-292-9820, fax: 787-522-2150; email: eduardo.nicolau@upr.edu

SUPPORTING INFORMATION

CB Particles and CBEMs

According to the manufacturer, BPs have a larger particle size than the VXC by a ten-fold. The oil absorption number (OAN) is a measure of the ability of a CB to absorb liquids; this property is the result of the structure of the CBs[1] and can influence the overall dispersion and agglomeration with organic solvents. The VXC has an OAN of 174 cc/100g, followed by the BP2 with 121 cc/100g, and the lowest value is the BP4 with 72 cc/100g. Lastly, the trend in densities was inversely to the one in OAN: VXC (264 kg/m^3) < BP2 (365 kg/m^3) < BP4 (375 kg/m^3).

Table S1. Comparison of the CB reported properties[1], and the experimental results of the BET surface area and water flux.

Studied CB	OAN (cc/100g)*	Density (kg/m^3)*	BET surface area (m^2/g):	Water Flux (LMH ± SD):
Vulcan (VXC)	174	264	221.8606	20.2 ± 4
Black Pearls 280 (BP2)	121	365	42.1984	14.2 ± 11
Black Pearls 430 (BP4)	72	375	77.0430	37.4 ± 14

The specific surface area of the untreated carbon blacks (CBs): VXC, BP2 and BP4 was determined by applying the Brunauer−Emmett−Teller (BET) method. To do so, the measurements were conducted using a TriStar II 3020. The degassing temperature was set at 80°C for 8 h under a N_2. As shown in **Table S1**, the VXC was the CB with highest surface area (221.8606 m^2/g). This was the main reason for being selected as the additive to immobilize at the membrane’s active layer.

SUPPORTING INFORMATION

Supports and Membrane Characterizations

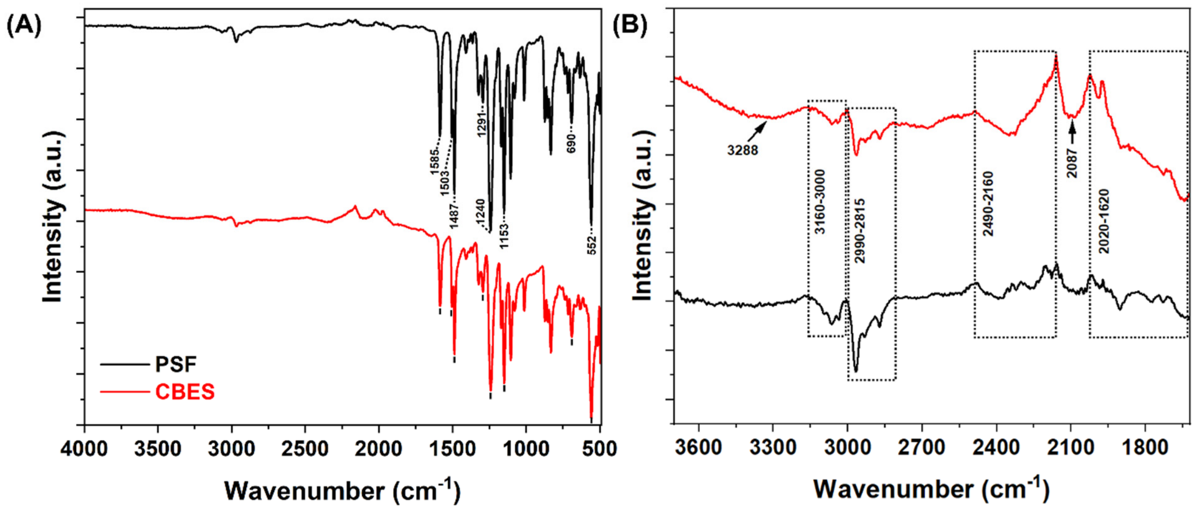

Figure S1. ATR-FTIR spectra of the fabricated supports **(A)** PSF and **(B)** CBES

The chemical composition of the fabricated supports was studied via ATR-FTIR, and the spectra is shown in **Figure S1**. The characteristics peaks of PSF are shown, including the aromatic ring stretching at 1585, 1503 and 1487 cm^{-1}, the SO_2 antisymmetric and symmetric stretches, and scissoring at 1291, 1153, and 552 cm^{-1}, respectively. Also, the C-O-C stretch is present at 1240 cm^{-1}, and the C-S stretch is shown at 690 cm^{-1}.These peaks were also present in the CBES support.

After a closer examination on the region from 4000 to 1620 cm^{-1} (**Figure S1B**), the C-H stretching vibrations in aromatics and alkyl are present in both supports in the regions of 3160-3000 cm^{-1} and 2990-2815cm^{-1}, respectively. The CBES spectra shows the distinct O-H stretching band at 3288 cm^{-1}, confirming the incorporation of additional functional groups. Also, an increase in intensity in the regions of CO and CO_2 adsorption is noticeable in the CBES, region 2490 – 2160 cm^{-1} and peak at 2087cm^{-1}, respectively. This can suggest that the incorporated CB particles increase the membrane adsorption capacity. Lastly, the aromatic overtones in the region of 2020-1620 cm^{-1} are

more prominent in the CBES suggesting an increment in the PSF benzenes dipole moment due to the presence of additional –OH functional groups. **Table S2** summarizes these vibrations bands.

Table S2. Summary of the peak location of the infrared bands of the main chemical functional groups in the fabricated supports: PSF and CBES

Wavenumber (cm^{-1})	Functional Group/Assignment
3288	O-H stretch
3160 – 3000	C-H stretch in aromatic
2990 – 2815	C-H stretch in alkyl
2490 – 2160	CO adsorption
2087	CO_2 adsorption
2020 – 1620	Aromatic overtones
1585, 1503 and 1487	Aromatic ring stretching
1291	SO_2 antisymmetric stretch
1240	C-O-C stretch
1153	SO_2 symmetric stretch
690	C-S stretch
552	SO_2 scissoring

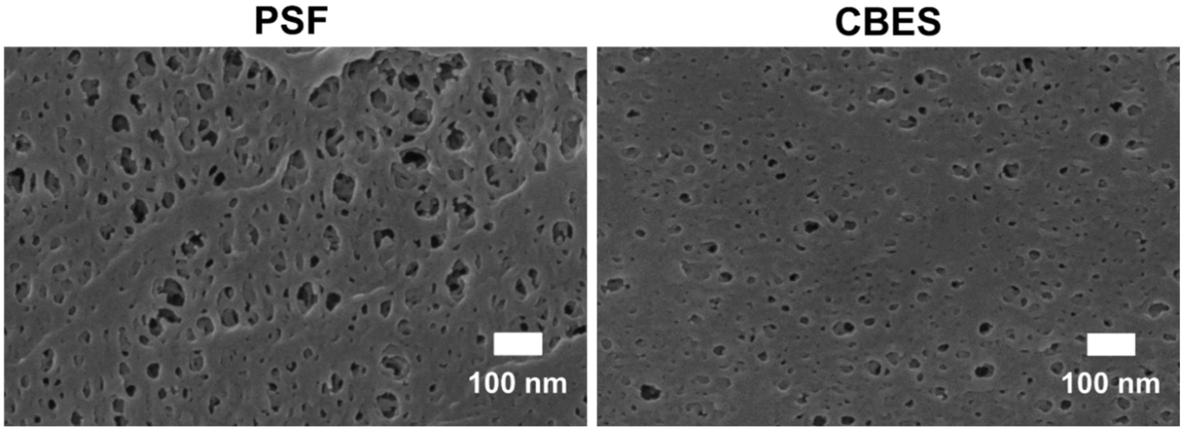

Figure S2. SEM micrograph at a 100,000x magnification

Figure S2 shows a closer examination of the support pores at their surface. In both cases the pores seem to be rounded and are distributed over all the surfaces. However, the pores in the CBES are smaller. The reduction in number of pores can be attributed to the CB capacity as nanofiller[2].

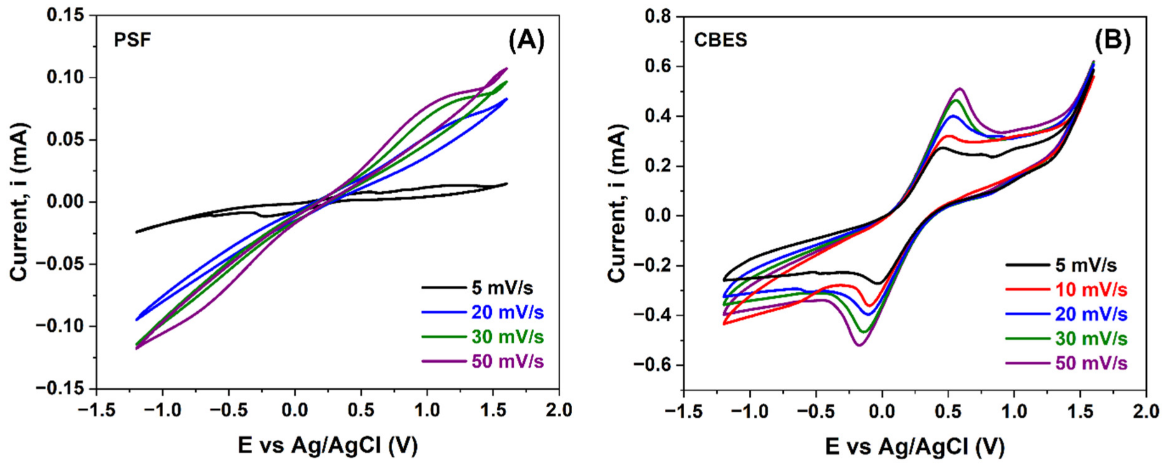

Figure S3. CV of Ferro/Ferricyanide at different scan rates (mV/s) using **(A)** PSF and **(B)** CBES as the working electrode.

In **Figure S3** the CV in the Ferri/Ferrocyanide probe are shown. The increment in scan rate reduces the surface diffusion layer and thus higher currents are reached. However, the electrochemical reversibility is slightly affected causing a larger ΔE.

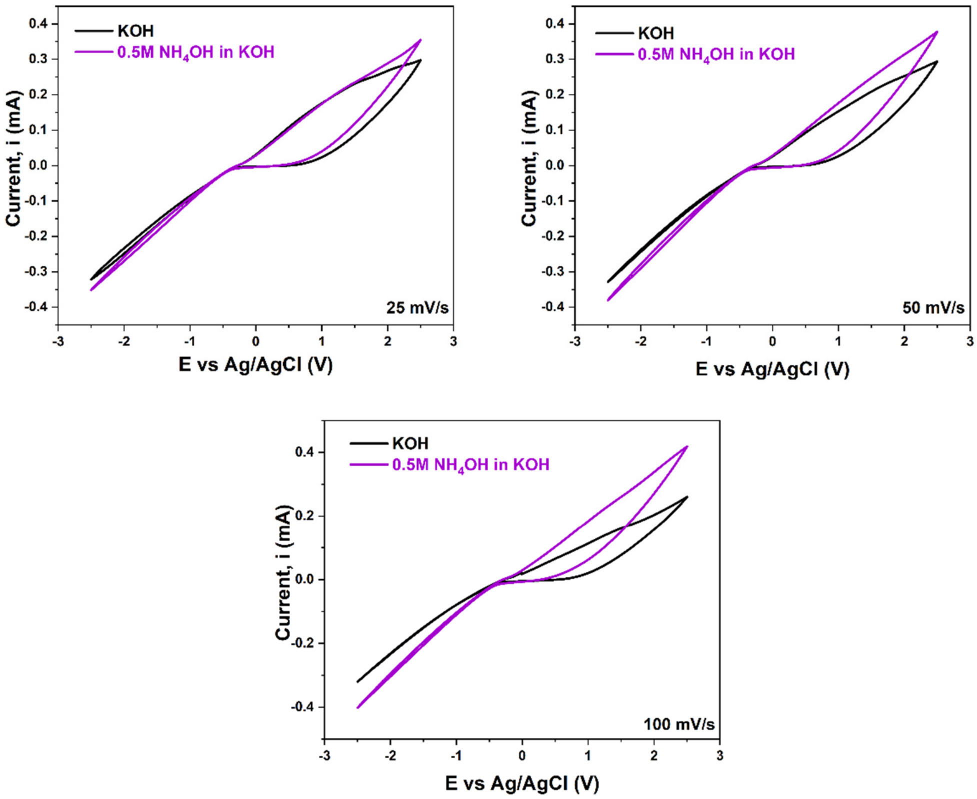

Figure S4. Ammonia electro-oxidation evaluation at different scan rates using CBES as the working electrode. Cyclic voltammograms were performed using 1M KOH as electrolyte. A Pt wire was used as counter electrode (CE) and Ag/AgCl (0.197V vs NHE) as the reference electrode (RE).

The voltammograms in **Figure S4** showed that the CBES were not able to complete the ammonia oxidation reaction (AOR). The CV were performed in a larger potential window to corroborate that the reaction was not present with a higher overpotential. The results indicate that the variation in scan rate did not affect the electrochemical response because similar anodic currents were obtained at the different scan rates. Therefore, even though the embedded CB enhance the

membrane electrochemical response are not sufficient to complete the charge-transfer reaction of the ammonia oxidation.

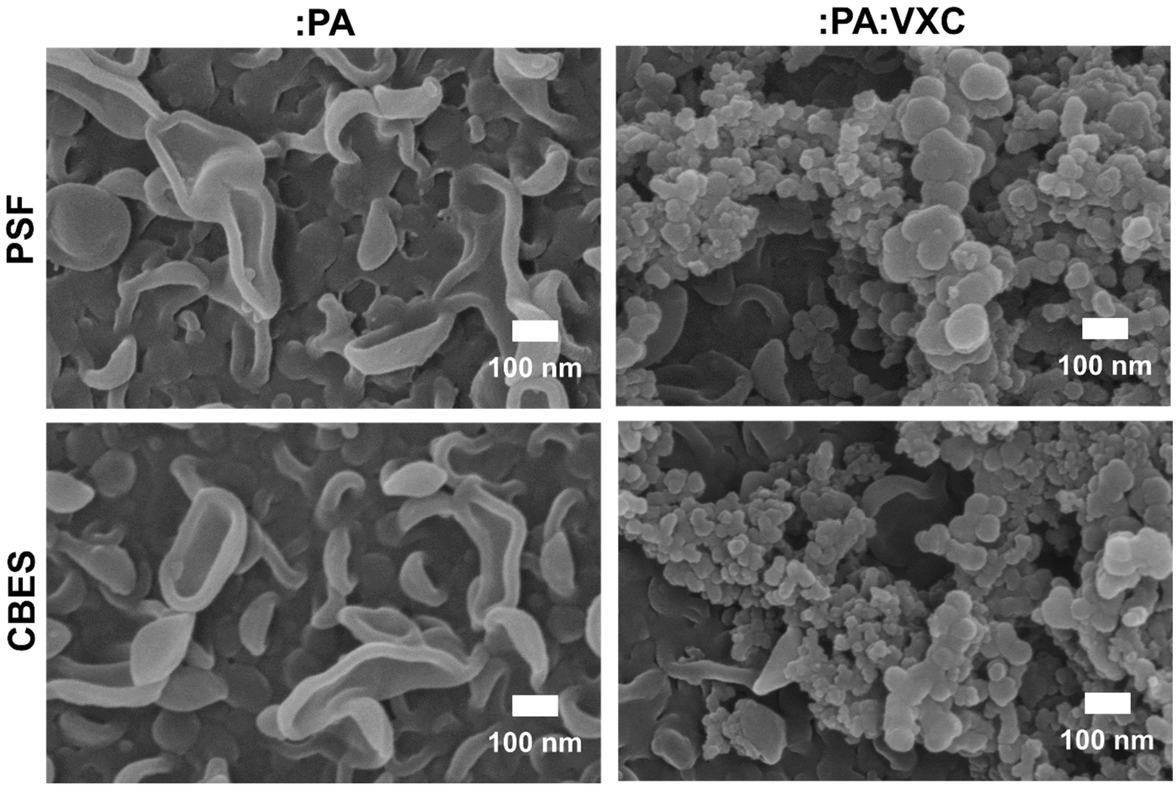

Figure S5. SEM micrographs at higher magnifications of the active layers: PA and PA:VXC over PSF and CBES

A closer examination of the :PA and :PA:VXC layers over both supports, suggests that the support chemical composition does not alter the morphological appearance of the studied materials. In **Figure S5** the typical PA morphology is shown in both supports, as well as a granular morphology for the VXC layer.

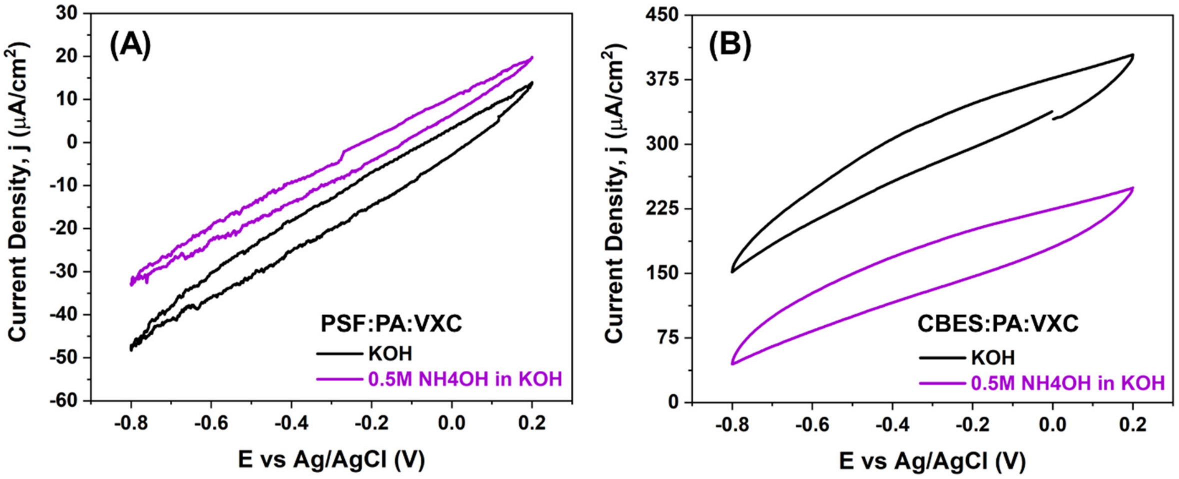

Figure S6. Ammonia electro-oxidation evaluation of **(A)** PSF:PA:VXC and **(B)** CBES:PA:VXC. Cyclic voltammograms were performed using 1M KOH as electrolyte. A Pt wire was used as counter electrode (CE) and Ag/AgCl (0.197V vs NHE) as the reference electrode (RE).

The :PA:VXC membranes have a small membrane impedance and a good overall electroactive surface. However, similar to the results in **Figure S4** the AOR was not successful (shown in **Figure S6**). This suggests that this type of reaction requires an additional electrocatalyst with selective sites for the reaction to take place.

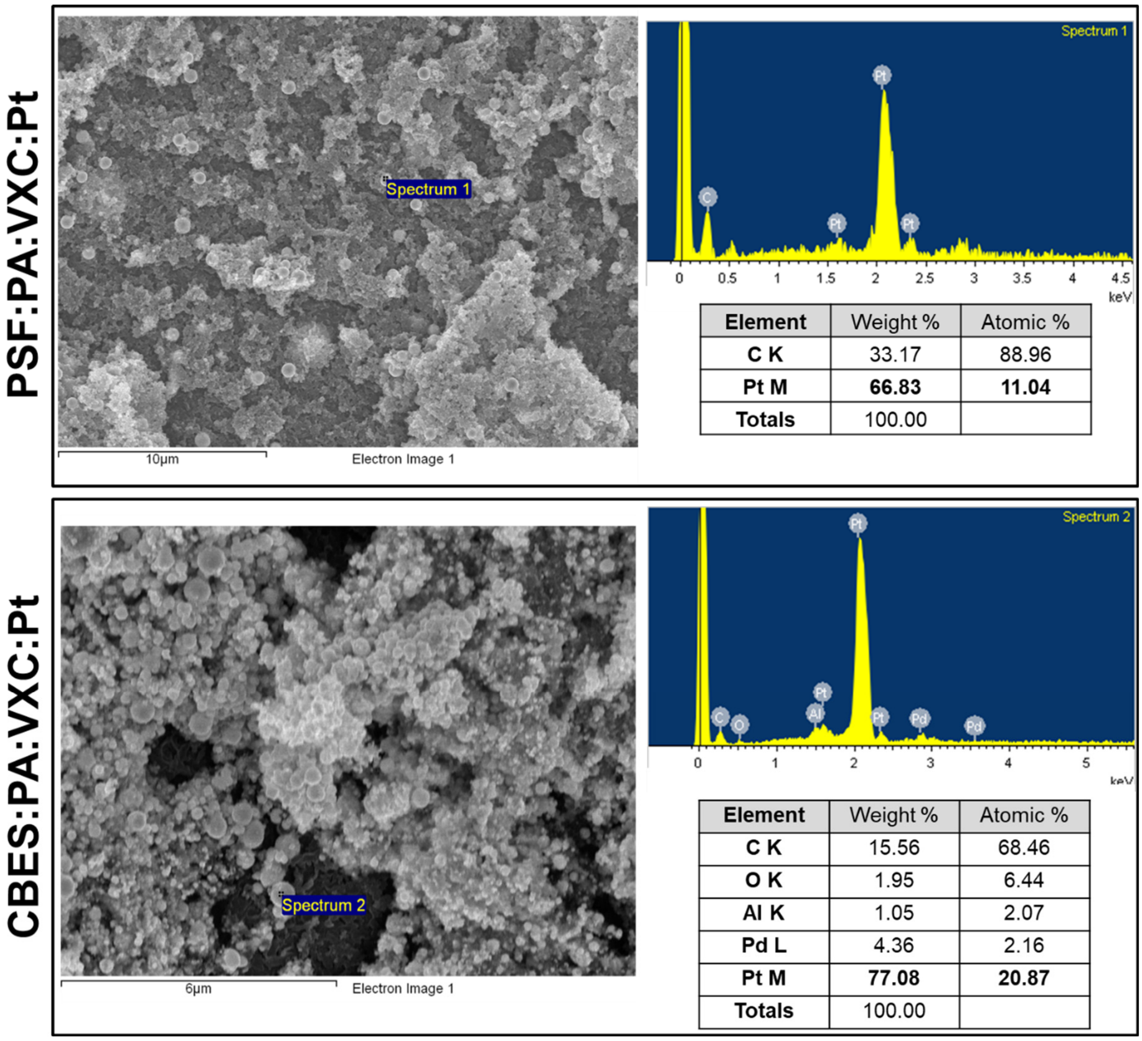

Element	Weight %	Atomic %
C K	33.17	88.96
Pt M	**66.83**	**11.04**
Totals	100.00	

Element	Weight %	Atomic %
C K	15.56	68.46
O K	1.95	6.44
Al K	1.05	2.07
Pd L	4.36	2.16
Pt M	**77.08**	**20.87**
Totals	100.00	

Figure S7. EDS results of both multilayered membranes: PSF:PA:VXC:Pt and CBES:PA:VXC:Pt

The EDS results in **Figure S7** were collected by analyzing some of the visible particles at the membrane surface. The tabulated results showed a high weight % of Pt in both membranes (66.8% in PSF:PA:VXC:Pt and 77.1% in CBES:PA:VXC:Pt). Confirming that the particles over the membrane supports are composed of platinum.

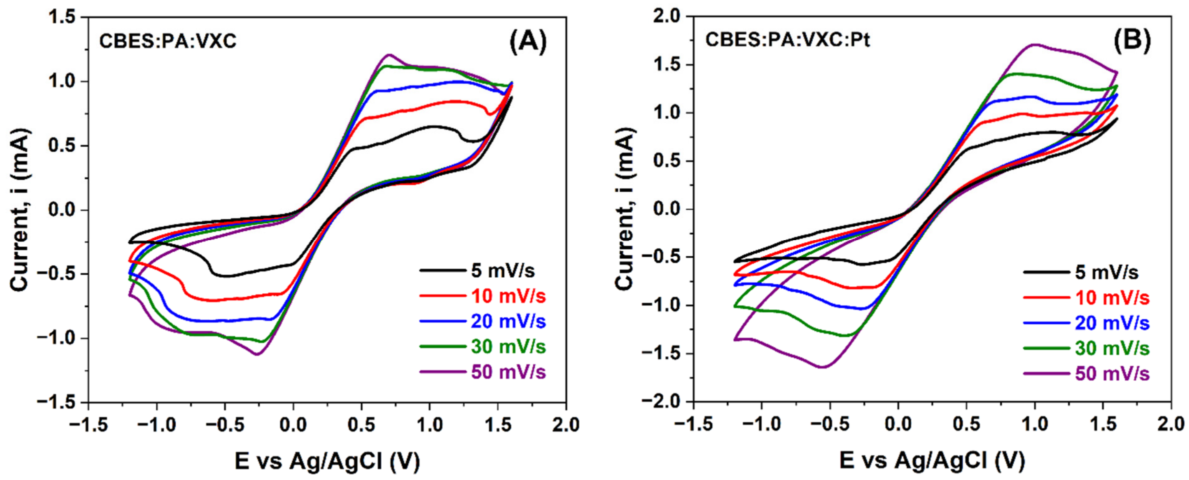

Figure S8. CV of Ferro/Ferricyanide at different scan rates (mV/s) using **(A)** CBES:PA:VXC and **(B)** CBES:PA:VXC:Pt as the working electrode.

In **Figure S8** we included the voltammograms where the Ferri/Ferrocyanide probe was monitored at different scan rates over the CBES:PA:VXC and CBES:PA:VXC:Pt membranes. The reversible one-electron reaction was observed. However, a larger current was reached in the presence of the PtNPs which confirms a more reactive surface.

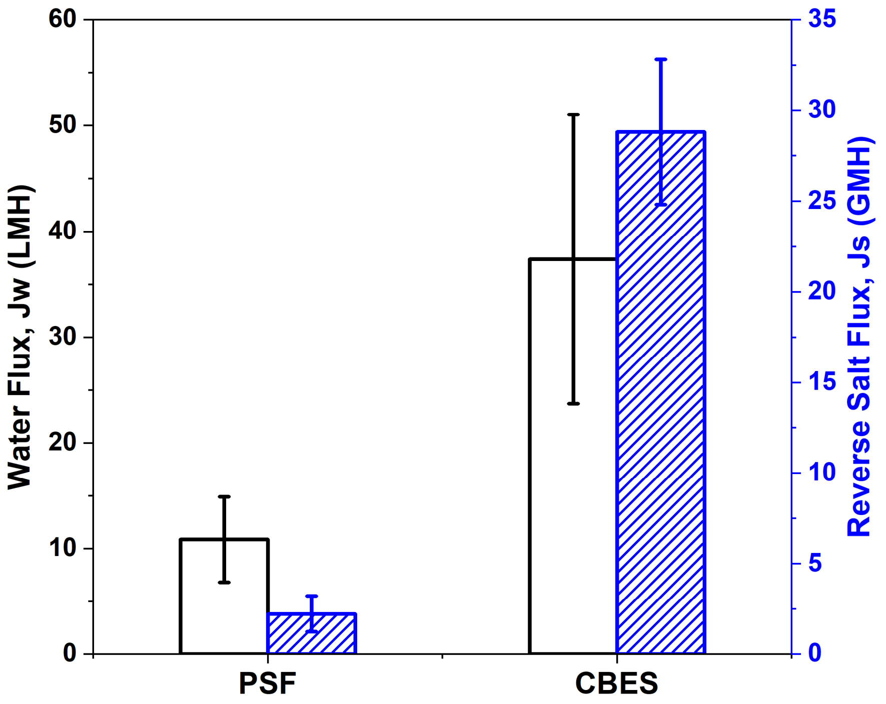

Figure S9. Water permeability and reverse salt flux comparison of the PSF and CBES

The water flux and reverse salt flux of the pristine supports are shown in **Figure S9**. A significant (71%) increase in water flux was observed when the CB particles were embedded in the support. However, due to the higher porosity and ion mobility ability of the CB particles, a high reverse salt flux was also noticed.

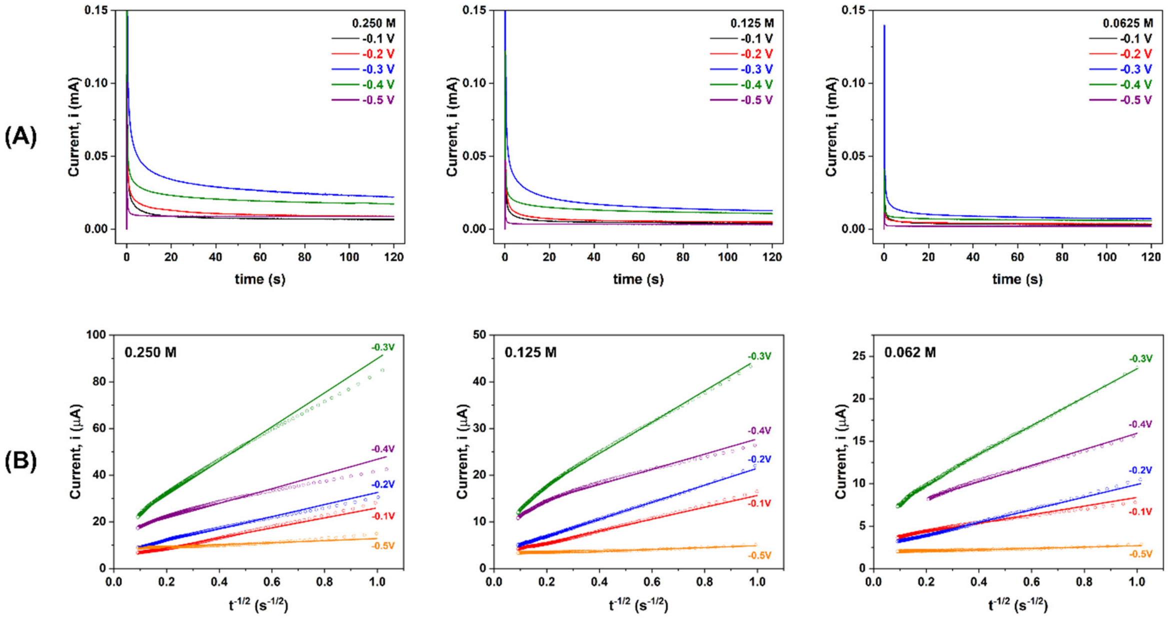

Figure S10. AOR activity measured at different potentials -0.1 V to -0.5 V vs Ag/AgCl and at different ammonia concentrations (0.250 M, 0.125 M and 0.0625 M). **(A)** Chronoamperometry measurements and **(B)** Cottrell plots.

The results in **Figure S10** corroborate the applied potential and ammonia concentration dependence on the AOR activity over the CBES:PA:VXC:Pt membrane. The Cottrell analysis plotted in **Figure S10B** confirmed that this reaction is diffusion-controlled and confirmed that the oxidation was conducted at -0.3 V vs Ag/AgCl. **Figure S11** compares the current decay when the concentration of ammonia was varied.

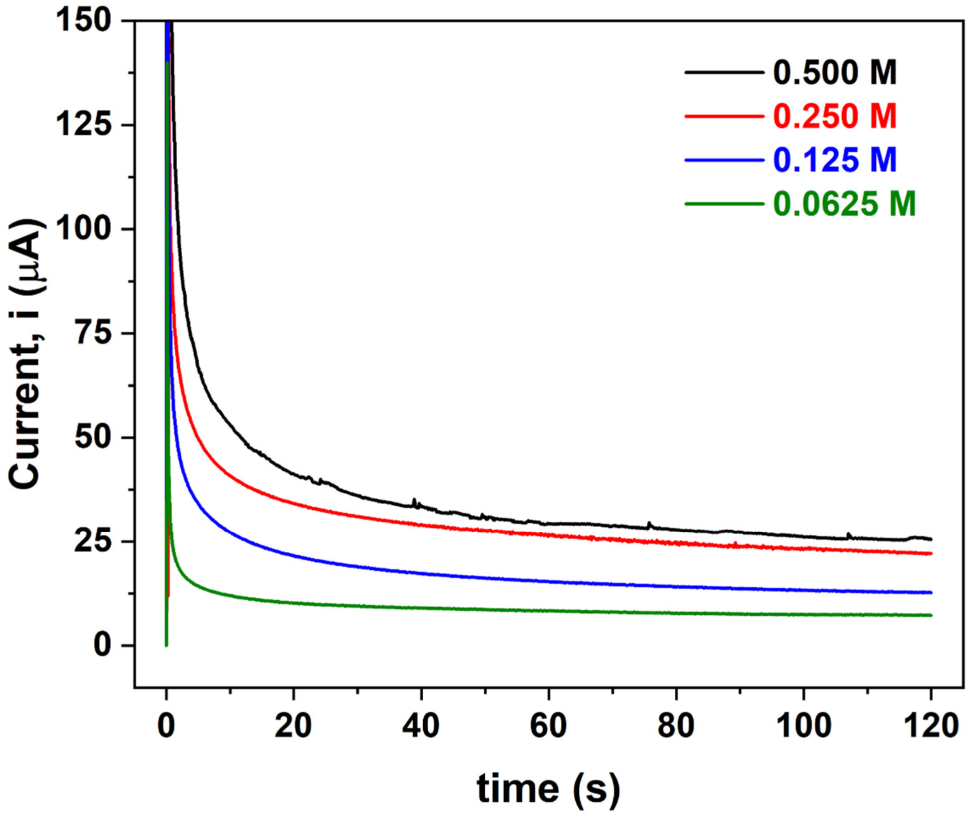

Figure S11. Evaluation of the AOR activity at constant -0.3 V vs Ag/AgCl potential while varying the NH_4OH concentration.

References

1. 4656:2012, I. Rubber compounding ingredients — Carbon black
2. Ray, S. S.; Gandhi, M.; Chen, S.-S.; Chang, H.-M.; Dan, C. T. N.; Le, H. Q., Anti-wetting behaviour of a superhydrophobic octadecyltrimethoxysilane blended PVDF/recycled carbon black composite membrane for enhanced desalination. *Environmental Science: Water Research & Technology* **2018,** *4* (10), 1612-1623.
